# Two‐Phase Inpatient Withdrawal Programme for Long‐Term Opioid Use in Non‐Cancer Pain

**DOI:** 10.1002/ejp.70010

**Published:** 2025-03-21

**Authors:** Konrad Streitberger, Michael A. Harnik, Anna Saliba, Nina Bischoff, Larissa T. Blättler, Kyrill Schwegler, Christine Baumgartner, Nora Sutter, Maria M. Wertli

**Affiliations:** ^1^ Department of Anaesthesiology and Pain Medicine, Inselspital, Bern University Hospital University of Bern Bern Switzerland; ^2^ Psychosomatic Medicine, Department of Neurology, Inselspital, Bern University Hospital University of Bern Bern Switzerland; ^3^ Department of Internal Medicine Kantonsspital Baden Baden Switzerland; ^4^ Department of General Internal Medicine, Inselspital, Bern University Hospital University of Bern Bern Switzerland

**Keywords:** chronic non‐cancer pain, chronic pain, inpatient withdrawal programme, opioid tapering, opioid treatment

## Abstract

**Background:**

High‐dose long‐term opioid treatment for chronic non‐cancer pain (CNCP) has become an increasing burden in industrialised countries. Opioid tapering and withdrawal in patients with CNCP remain challenging. This study evaluated a two‐phase inpatient opioid withdrawal (OW) programme aimed at safely discontinuing opioid use in CNCP patients.

**Methods:**

This prospective observational study was conducted from 2018 to 2023 at a Swiss tertiary care centre, involving CNCP patients on long‐term opioid therapy (≥ 6 months, ≥ 100 mg morphine equivalent daily dose) who had failed outpatient withdrawal attempts. The programme consisted of a withdrawal phase (Phase 1) followed by multimodal pain rehabilitation (Phase 2). Outcomes included the proportion of patients opioid‐free after Phase 2 (primary) and at 3 months, pain intensity changes, and adverse events (secondary).

**Results:**

Among the 38 enrolled patients (58% female, median age 54 years [IQR 49, 62]), 34 (89%) completed both phases, and 32 (84%) were opioid‐free at the end of Phase 2. At 3 months, 23 patients (61%) remained opioid‐free, while 4 (11%) resumed opioids, and 11 (29%) were lost to follow‐up. Median pain intensity remained stable after discharge. One patient died by suicide 10 days post‐withdrawal.

**Conclusions:**

This two‐phase inpatient withdrawal and rehabilitation programme enabled most CNCP patients to discontinue opioids without increased pain intensity, with a majority remaining opioid‐free at 3 months. These findings highlight the importance of ongoing psychological support and careful patient selection in OW management.

**Significance Statement:**

This study introduces a structured inpatient opioid withdrawal model tailored for chronic non‐cancer pain patients on high‐dose opioid therapy, demonstrating that high cessation rates can be achieved without worsening pain intensity. By addressing the gap in care for patients who fail outpatient tapering, this research provides clinical insights into optimising withdrawal protocols and highlights the need for targeted resource allocation for intensive, multidisciplinary pain management. These findings support evidence‐based decision‐making in designing more effective opioid tapering strategies.

## 1 Introduction

The prescription of opioids for chronic non‐cancer pain (CNCP) has significantly contributed to the global opioid crisis, particularly in the United States, where opioid‐related fatalities have surged, resulting in over 16,000 deaths annually (Els et al. 2023; Vowles et al. 2015). The number of overdose deaths involving opioids has increased nearly fivefold between 1999 and 2021 (Atlanta 2021).

In Europe, it is estimated that approximately 19% of the population experiences moderate‐to‐severe CNCP, with approximately two‐thirds taking prescription medications to manage pain (Breivik et al. 2006). This trend was reflected in a systematic review of 60 studies across various regions, including the United States, South America, Australia, Europe, and Israel, where approximately one‐fourth of individuals with CNCP reported opioid use (Wertheimer et al. 2021). Despite national guidelines discouraging opioid prescriptions for CNCP (Busse et al. 2018; Carville et al. 2021; Häuser et al. 2020), recent studies conducted in Switzerland have shown an increase in prescriptions for weak (+13%) and strong (+121%) opioids between 2006 and 2013 (Wertli et al. 2017) and a significant increase in strong opioids (+88.4%) for minor and major musculoskeletal injuries between 2008 and 2018 (Müller et al. 2023). In Switzerland, despite not facing the same crisis as the U.S., opioid prescriptions have risen significantly, necessitating closer examination and intervention (Hooijman et al. 2022).

A systematic review of seven international guidelines recommended discontinuation of opioid prescriptions when harm outweighs the benefits, that is, when substantial improvement in pain intensity or function is lacking (Hamilton et al. 2023). However, tapering can be challenging in an outpatient setting because of the risk of withdrawal symptoms, cravings, and suicide. Therefore, opioid withdrawal is considered a high‐risk intervention (Forget 2022; Hallvik et al. 2022; Kurita et al. 2018.). If gradual outpatient tapering is not feasible, an inpatient setting is recommended (Häuser et al. 2020). A systematic review found that interdisciplinary pain programmes achieved an average opioid discontinuation rate of 87%, with rates ranging from 29% to 100% across outpatient and inpatient settings. Among 10 studies that required discontinuation as a condition of enrolment, success rates ranged from 74% to 100% (Frank et al. 2017). While various interventions have been studied, their comparative effectiveness in reducing or discontinuing long‐term opioid therapy remains unclear. Moreover, the benefit–risk ratio for opioid tapering or discontinuation remains unclear, given that serious harm such as substance use, opioid overdose, and suicide can occur (Mackey et al. 2020; Avery et al. 2022).

Our objective was to describe a two‐phase inpatient opioid withdrawal (OW) programme and to assess the number of opioid‐free CNCP patients at two points in time: immediately and 3 months after OW. Furthermore, we aimed to assess the change in the average pain intensity before and after OW, and possible adverse outcomes were monitored. The hypothesis was that CNCP patients receiving high‐dose long‐term opioid treatment (LTOT) can be successfully and safely withdrawn in an inpatient setting without a significant increase in pain and no harmful negative impact.

## 2 Methods

This study was an analysis of prospectively collected quality control data from a newly implemented two‐phase OW programme at a Swiss tertiary care centre. The programme was designed as a clinical quality improvement initiative to evaluate and optimise outcomes for patients undergoing structured OW. Data collection was part of routine clinical documentation and adhered to quality management protocols. Literature supports the effectiveness of combining OW with multimodal pain management for CNCP patients (Gatchel and Okifuji 2006; Turk and K 2007). Consequently, the study was structured into two distinct phases: Phase 1 focused on physical withdrawal, and Phase 2 involved a comprehensive multimodal pain rehabilitation programme.

### 2.1 Study Population

Patients on long‐term opioid treatment were either referred for OW by their family doctors or recruited from outpatient clinics. Eligibility was assessed by an interdisciplinary team (specialists in pain medicine, general internal medicine, and psychosomatic medicine). Inclusion criteria were age ≥ 18 years with persistent opioid use (≥ 6 months) at a daily dose of ≥ 100 mg morphine equivalent (MED). Furthermore, patients had to show at least one of the following features:

1. Relative ineffectiveness of opioid therapy, defined as insufficient pain relief despite high‐dose opioid therapy, indicated by a Numeric Rating Scale (NRS) score of > 5 (scale 0–10) over a period of at least 3 months.
2. Recurrent dose escalation is defined as repeated dose increases of at least 50% since the initiation of opioid therapy.
3. Intolerable opioid‐related side effects (e.g., cognitive disturbances, dysphoria, loss of drive and motivation, persistent nausea, severe constipation, and visual disturbances).
4. Signs of opioid‐induced hyperalgesia or central sensitisation (measured by ALGOPEG (Egloff et al. 2011)) (Angst and Clark 2006).
5. Signs of opioid dependence and abuse, evaluated using the Current Opioid Misuse Measure (COMM) questionnaire, administered at a single point during the initial assessment, with a score of ≥ 9 indicating elevated risk (Butler et al. 2007).

Furthermore, patients needed to be highly motivated to stop opioid treatment, a criterion primarily evaluated through direct discourse during interdisciplinary clinical assessment.

The exclusion criteria were myocardial infarction, pulmonary embolism, stroke within the last 6 months, planned surgery in the foreseeable future, and insufficient language proficiency in German or French, which prevented patients from understanding the programme and following instructions. Other reasons for exclusion were pregnancy or breastfeeding, as well as evidence of severe psychiatric conditions. These were carefully explored in collaboration with a psychiatrist, and patients were not included in the programme when deemed too hazardous (e.g., bipolar disorder, schizophrenia or other psychotic disorders, addiction and substance abuse other than to opioids, and major depression with or without suicidal tendencies).

All the patients included in the OW were asked to participate in the cohort study as part of a quality control programme. This study was approved by the local ethics committee (Ethics Committee of the Canton of Bern; KEK ID 2020‐01226).

### 2.2 Inpatient Opioid Withdrawal (Phases 1 and 2)

Due to the resource‐intensive nature of the programme, enrolment was limited to one patient at a time, requiring individualised care and substantial staff involvement. OW was conducted following a multidisciplinary management approach: During Phase 1, opioid doses were systematically reduced using a stepwise protocol developed at the Pain Centre of Bochum University Hospital, Germany (Bienek et al. 2019; Krumova et al. 2013). Initially, opioid doses were converted into morphine equivalents (mg MED) using standardised conversion tables (Plagge et al. 2011). Patients were transitioned to oral morphine solution, starting with an initial dose of 100 mg MED, divided into six doses administered every 4 h to maintain stable plasma levels. The liquid formulation allowed precise dose titration and facilitated gradual reductions.

The morphine dose was reduced by 10–20 mg every second day until a daily dose of 10 mg was reached, after which morphine was discontinued. Adjustments to the reduction schedule were made based on individual tolerance and withdrawal symptoms (see Table S1 for the detailed stepwise reduction protocol).

The choice of liquid morphine was made for maintaining flexibility during withdrawal. Regular administration ensured steady plasma levels, reducing the risk of withdrawal symptoms. Furthermore, switching patients from various opioids to morphine served as an opioid rotation strategy, which has been shown to improve therapeutic effectiveness by reducing opioid tolerance and hyperalgesia (Fine and Portenoy 2009).

Patients were thoroughly informed about the reduction procedure, potential withdrawal symptoms, and available alternative analgesics. All patients underwent ear acupuncture following the National Acupuncture Detoxification Association (NADA) protocol (Litscher 2019) and were pre‐treated with a minimum dose of 75 μg clonidine to alleviate withdrawal symptoms. Withdrawal management also included medications such as quetiapine, benzodiazepines (lorazepam or diazepam), or tricyclic antidepressants as needed. Non‐pharmacological interventions were integral to the individualised treatment plan (Table 1).

**TABLE 1:** Overview of non‐pharmacological interventions during opioid withdrawal.

Intervention	Explanation	Frequency
Acupuncture	Ear acupuncture following the National Acupuncture Detoxification Association protocol	Permanent needles throughout opioid withdrawal
Education and information	Information provided by medical staff about the withdrawal process, symptoms, and available therapies	Daily short contacts during both phases
Mindfulness‐based stress reduction (MBSR)	Acceptance and commitment based therapy	Weekly group session in Phase 2
Cognitive‐behavioural therapy	Psychological support focused on pain and stress management	Individual support during opioid withdrawal and weekly group and individual sessions in Phase 2
Physiotherapy	Includes active therapy, body awareness therapy, Nordic walking, transcutaneous electrical nerve stimulation, and bath therapy	Daily group therapy and weekly individual sessions in Phase 2; individualised sessions during withdrawal
Relaxation techniques	Includes progressive muscle relaxation, breathing exercises, body scan, and biofeedback	Weekly sessions in Phase 2
Occupational therapy	Focused on posture training and coping with everyday life	Weekly group sessions in Phase 2
Routine nursing applications	Relaxation‐focused interventions such as foot baths or embrocation	Every evening if desired
Social support	Assistance with professional and financial concerns provided by social workers	When indicated

In cases of refractory pain, continuous intravenous ketamine was administered, with doses titrated up to a maximum of 30 mg/h under routine monitoring in the ward. Phase 1 concluded when the patient had been opioid‐free for at least 24 h and withdrawal symptoms were adequately controlled through conservative management.

Following Phase 1, patients were discharged home for a 3‐day period before commencing Phase 2, a 3‐week interdisciplinary multimodal pain rehabilitation programme conducted in the psychosomatic ward. This phase of treatment incorporated physical and occupational therapy, individual and group psychotherapy based on cognitive‐behavioural principles to facilitate self‐management of mood and pain, PMR, mindfulness, and other mind–body techniques (Table 1) (Arnold et al. 2009). The overarching goal was to foster a multidisciplinary approach to chronic pain management and empower patients to take an active role in their care. Conventional pain medications were administered as required. Upon discharge from Phase 2, opioid withdrawal was deemed complete, and subsequent steps, including outpatient follow‐up care, were discussed with each patient. If additional disorders requiring treatment were identified, appropriate diagnostic workup and treatment were arranged.

### 2.3 Measures and Outcomes

Prior to entering the programme, all patients completed a series of validated self‐report questionnaires as part of the standard evaluation at the hospital's outpatient pain clinic. Pain intensity was measured using an NRS 0–10, where 0 represents “no pain” and 10 represents the “worst imaginable pain”. As recommended by the BPI, all four items of the BPI were recorded (minimum, maximum, average pain and pain now) (Cleeland 2009). Further questionnaires included the Pain Catastrophizing Scale (PCS; range: 0–52) to assess catastrophic thinking related to pain (Sullivan et al. 1995), the Multidimensional Pain Inventory (MPI; range: 0–150) to evaluate the psychosocial impact of chronic pain (Kerns et al. 1985), and the Beck Depression Inventory (BDI; range: 0–63) to assess depressive symptoms (Beck 1961). Demographic and clinical data were extracted from electronic medical records.

Patients were contacted 3 months post‐discharge and asked to complete a follow‐up questionnaire, which included the Patient Global Impression of Change (PGIC) to capture patients' perceived improvement in pain and overall situation (Hurst and Bolton 2004). If no response was received, the questionnaire was sent again. Patients were considered lost to follow‐up after three unsuccessful contact attempts via phone and email. Tolerance of the OW process was evaluated by asking patients a single question: “Would you again undergo the programme to stop opioid treatment?” Additionally, patients were asked to report any serious adverse events. This study adheres to the STROBE statement for reporting (von Elm et al. 2014).

The primary outcome was the proportion of patients who successfully stopped opioid treatment upon completion of Phase 2. Secondary outcomes included the proportion of patients who remained opioid‐free after 3 months and the change in average pain intensity from baseline (measured as average pain at the start of Phase 1) to discharge after Phase 2. Average pain was selected as a global measure of pain intensity due to its simplicity and established use in clinical pain research (Cleeland 2009). We also evaluated the proportion of patients who achieved an improvement of ≥ 2 points on the NRS for average pain intensity between baseline and discharge, a change considered to be the minimal clinically important difference (MCID) (Kamper et al. 2009; Salaffi et al. 2004). Reasons for discontinuation of the programme, if applicable, were documented.

### 2.4 Statistical Analysis

Since patient data were collected as part of quality control, no formal sample size calculation was performed. The number of cases was limited by the programme's treatment capacity, which allowed for up to 15 patients per year. All patients with informed consent were included in the outcome analysis, and missing data were considered treatment failures using an intention‐to‐treat approach. For categorical variables, we calculated the percentage of patients in each category, and for continuous variables, we computed the median along with the first (Q1) and third quartiles (Q3).

Unadjusted group comparisons of categorical variables were performed using Pearson's chi‐square test. Changes in average pain intensity over the three timepoints (baseline, after Phase 2, and after 3 months) were assessed using the Friedman test for participants with complete data across all timepoints. Post hoc pairwise comparisons were performed using Dunn's test with Bonferroni correction to control for multiple comparisons. All computations were performed using R, version 4.0.2 (R Core Team 2020), and statistical significance was set at *p* < 0.05.

## 3 Results

### 3.1 Study Cohort and Patient Characteristics

**FIGURE 1:**
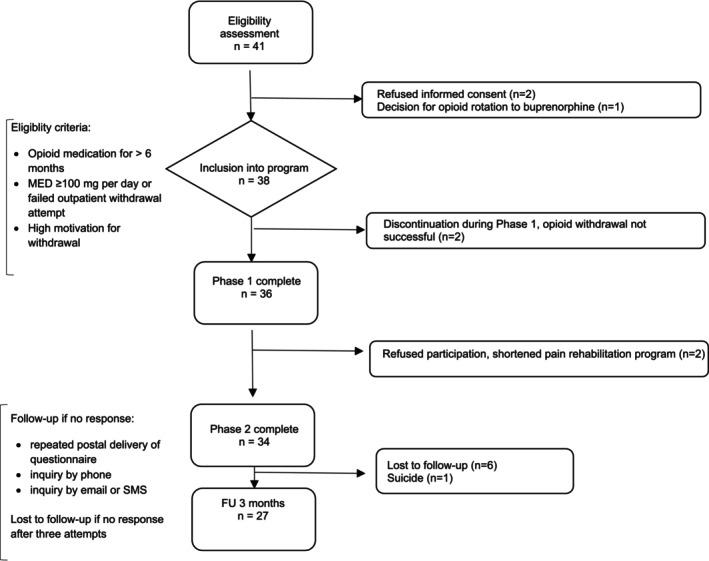
Flowchart describing the inclusion, progression, and follow‐up phases of the withdrawal programme. The diagram details patient eligibility, inclusion in the programme, and reasons for discontinuation across the two phases, as well as follow‐up outcomes.

A total of 41 patients were evaluated for the programme between March 2018 and March 2023. Two patients declined to provide informed consent, and one candidate with a palliative non‐cancer diagnosis was scheduled for opioid rotation instead of withdrawal. The remaining 38 patients were enrolled in the OW programme and provided consent for inclusion in the cohort study (Figure 1). More than half of the patients were female (22 patients, 58%), with a median age of 54 years [Q1, Q3: 49, 62] (Table 2).

**TABLE 2:** Demographic and clinical characteristics of the group.

	Overall, *n* = 38	Female, *n* = 22 (58%)	Male, *n* = 16 (42%)
Age (years)	54 [49, 62]	53 [47, 62]	57 [51, 63]
BMI (kg/m^2^)	26.1 [23.4, 29.8]	25.0 [21.9, 28.3]	28.0 [24.2, 30.0]
Opioids			
Morphine	5 (13%)	3 (14%)	2 (13%)
Fentanyl	13 (34%)	10 (45%)	3 (19%)
Hydromorphone	1 (3%)	1 (5%)	0 (0%)
Pethidine	1 (3%)	1 (5%)	0 (0%)
Oxycodone	17 (45%)	6 (27%)	11 (69%)
Buprenorphine	2 (5%)	1 (5%)	1 (6%)
Tapentadol	5 (13%)	4 (18%)	1 (6%)
Dosage form			
Extended release	17 (45%)	10 (45%)	7 (44%)
Short release	8 (21%)	5 (23%)	3 (19%)
Both	12 (32%)	6 (27%)	6 (38%)
Oral	28 (74%)	15 (68%)	13 (81%)
MED (mg)	180 [120, 263]	173 [120, 270]	180 [120, 225]
Pain duration (years)	9 [4, 16]	9 [4, 16]	10 [5, 18]

*Note:* Data are shown as median [Q1; Q3] for continuous or ordinal data and as absolute and relative frequencies (*n* %) for categorical data.

Abbreviations: BMI, body mass index; MED, morphine equivalent dose.

The indications for opioid treatment included chronic musculoskeletal pain (13 patients, 34%), postoperative pain (12 patients, 32%), neuropathic pain (11 patients, 29%) and complex regional pain syndrome (2 patients, 5%), that is ICD‐11 diagnoses MG 30.3 and 30.02, MG 30.2, MG 30.5, and MG 30.04, respectively (Treede et al. 2015). Minor depression was diagnosed in 18 patients (50%), and post‐traumatic stress disorder in three patients (8%). Oxycodone was the most commonly used opioid (17 patients, 45%), followed by transdermal fentanyl (13 patients, 34%). The median daily morphine equivalent dose (MED) on admission was 180 mg [120, 263].

### 3.2 Outcomes

**FIGURE 2:**
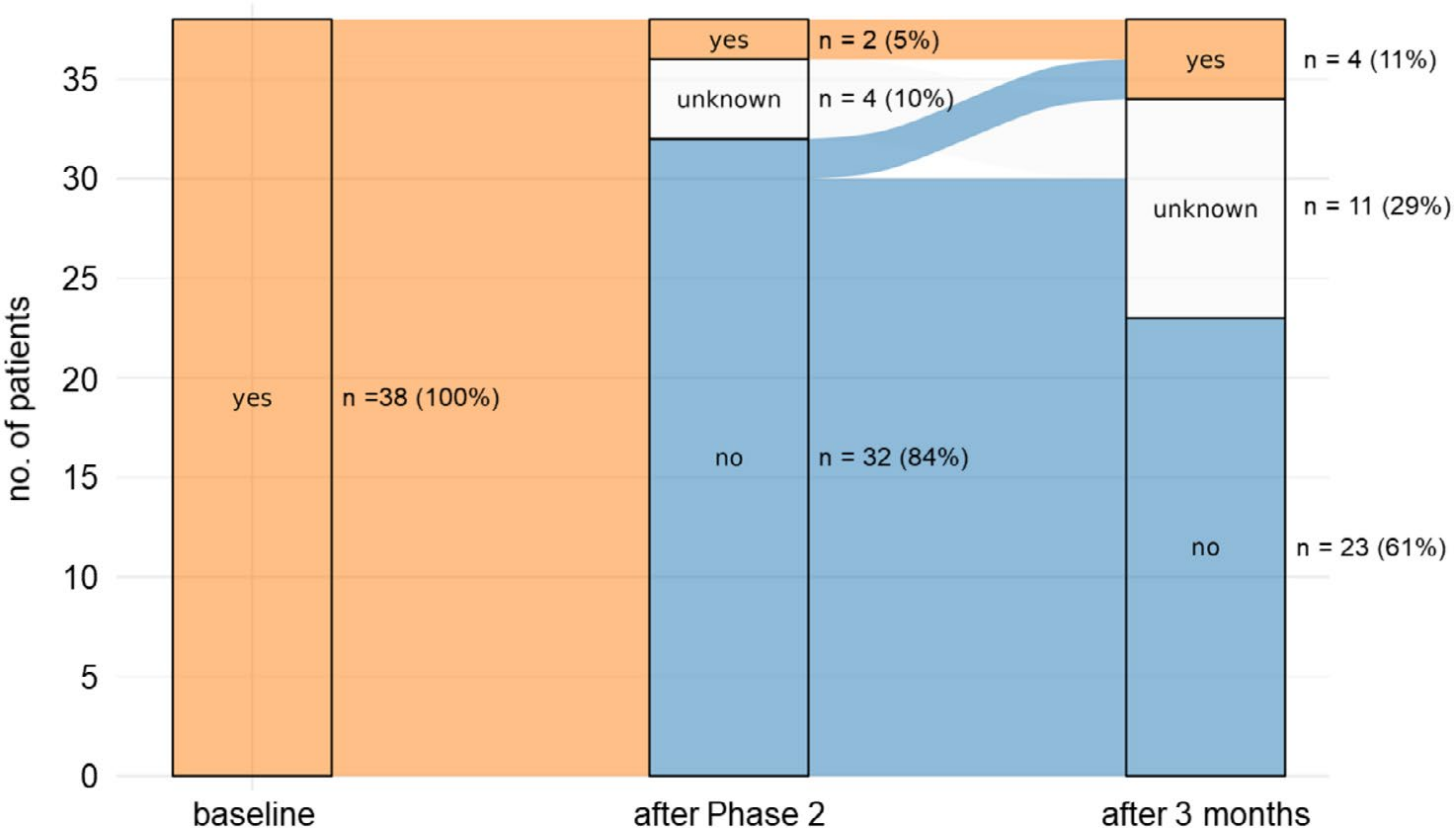
A significant proportion of patients remained opioid‐free after 3 months. Alluvial chart showing the flow of patients across three time points: Baseline, after Phase 2, and 3 months after the programme. The chart tracks the absolute and relative frequencies of opioid use at each stage. The *x*‐axis represents the timeline of the withdrawal programme, with three key time points: Baseline (before the programme), after Phase 2, and after 3 months (post‐programme follow‐up). The *y*‐axis represents the number of patients at each time point, ranging from 0 to 38.

Of the 38 patients included in the OW programme, 34 (89%) completed the programme as planned, and 32 (84%) were opioid free after Phase 2 (Figure 2).

**FIGURE 3:**
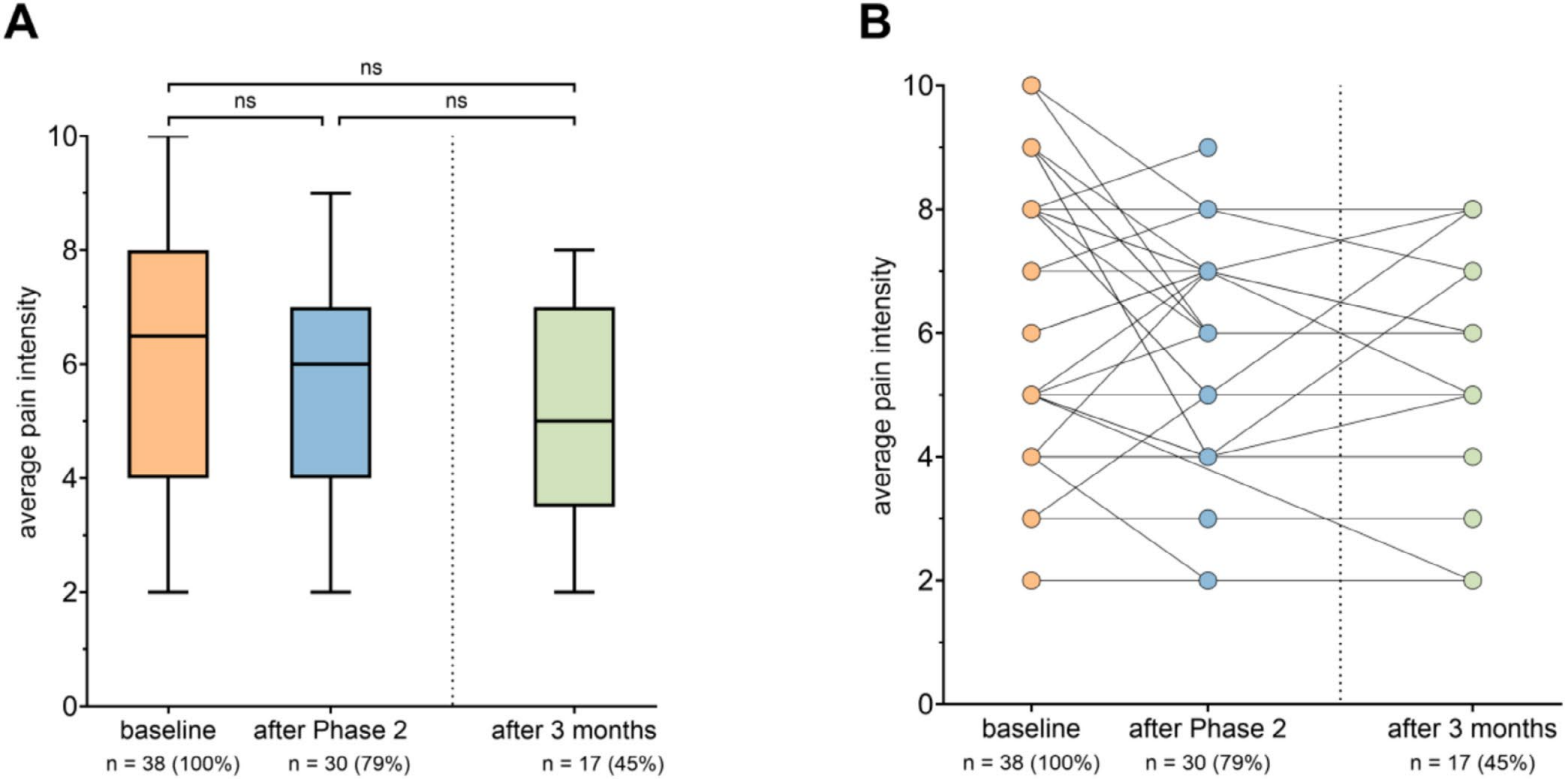
The average pain intensity did not significantly change after opioid withdrawal. (A) Boxplots showing average pain intensity at baseline, after Phase 2, and after 3 months for all participants with available data. Median [Q1; Q3] is shown; whiskers indicate minimum and maximum values. Statistical analyses (Friedman test with Dunn's post hoc test) were performed using a subset of participants with complete data across all timepoints (*n* = 15). *p*‐values from these tests refer only to this subgroup and are displayed for transparency. (B) Individual trajectories of average pain intensity for all participants with data at the respective timepoints.

Two patients were unable to discontinue opioids due to uncontrollable pain, necessitating adjustments in opioid doses and formulations to balance pain relief with side effects. None of the patients relapsed during the 3‐day period at home between the two phases. Compared to baseline, average pain intensity did not change significantly despite opioid cessation (baseline median NRS: 6.5 [4, 8]; after Phase 2 NRS: 6 [4, 7]; *p* > 0.99; Table 3 and Figure 3A).

**TABLE 3:** Outcome measures.

Variable	Baseline (*n* = observed patients)	After Phase 2 (*n* = observed patients)	After 3 months (*n* = observed patients)
Minimum pain (NRS, median [IQR])	4 [3; 6], (*n* = 18)	4 [3; 5.5], (*n* = 15)	4 [3; 5], (*n* = 17)
Average pain (NRS, median [IQR])	6.5 [4; 8], (*n* = 38)	6 [4; 7], (*n* = 30)	5 [4; 7], (*n* = 17)
Maximum pain (NRS, median [IQR])	8 [7; 9], (*n* = 19)	8 [6; 10], (*n* = 15)	8 [6; 9], (*n* = 17)
Current pain (NRS, median [IQR])	6 [5; 6.5], (*n* = 18)	5 [4.5; 8], (*n* = 15)	5 [3; 7], (*n* = 17)
MCID achieved, *n* (%)	—	11 (29%)	2 (5%)
Successful OW, *n* (%)	—	32 (84%)	23 (61%)
Would repeat OW, *n* (%)	—	24 (63%)	19 (50%)

*Note:* Data of all included patients (*n* = 38) are shown as median [Q1; Q3] for continuous or ordinal data and as absolute and relative frequencies (*n*, %). Observed patient numbers (*n*) are indicated for each variable and timepoint.

Abbreviations: MCID, minimal clinical important change of ≥ 2 points; NRS, numerical rating scale; OW, opioid withdrawal.

Among the 34 patients who completed the programme, 11 (29%) showed a reduction of 2 or more points on the NRS. Data were missing for eight patients (21%). Three participants (9%) experienced a worsening in pain (≥ 2 points on the NRS, with no participant having a development on the NRS that would have precluded a 2‐point deterioration; Figure 3B). After 3 months, 27 of the 34 patients (79%) responded to the follow‐up questionnaires, of whom 23 (61% of the total cohort and 85% of responders) remained opioid‐free. Most patients (19, 70%) stated that they would participate in the programme again. One patient committed suicide 10 days after discharge.

### 3.3 Additional Self‐Reported Outcomes

In addition to pain intensity, patients completed validated questionnaires assessing psychological and psychosocial factors, including the PCS, MPI, and BDI. The results show slight improvements in PCS and BDI scores, while MPI scores remained stable. Response rates varied across measures, with available data reported for transparency (Table S2). Given the limited sample size for these measures, statistical analyses were not performed.

The Patient Global Impression of Change (PGIC) at 3 months had the highest response rate among self‐reported outcomes (*n* = 20 for pain, *n* = 21 for overall condition) and provided the clearest subjective assessment of treatment effects. PGIC responses were mixed, with 50% of patients reporting an improvement in pain and 50% reporting no change or worsening. For overall condition, 67% of patients reported improvement, 19% reported no change, and 14% experienced worsening.

### 3.4 Reasons for Discontinuation of the Programme

During Phase 1, two patients discontinued the programme. One patient, with a history of childhood trauma, experienced increasing and intense anxiety, necessitating cessation of OW. The second patient, suffering from central neuropathic pain following a severe spinal injury, had to terminate OW due to intolerable pain despite ketamine administration, leading to the reinstatement of opioid therapy. During Phase 2, two additional participants opted to leave the programme: one due to persistent withdrawal symptoms such as diarrhoea, headaches, and irritability, and the other was required to discontinue the programme by the intervention team due to non‐compliance with hospital hygiene regulations during the COVID‐19 pandemic, specifically refusing to adhere to the mandatory mask policy. No follow‐up data were available for these individuals regarding opioid use.

## 4 Discussion

This study demonstrates the feasibility of a two‐phase OW programme for CNCP patients on LTOT. Most discontinued opioids without a significant increase in pain, with one‐third reporting clinically relevant relief. At follow‐up, 85% remained opioid‐free, and 70% were willing to repeat the programme. However, one patient's suicide underscores the need for robust follow‐up care.

### 4.1 Feasibility of Opioid Withdrawal Programme

Our protocol, modified after Krumova et al. 2013, used stepwise opioid reduction with pharmacological and non‐pharmacological support. Patients discharged between OW and multimodal rehabilitation showed no increased dropout or relapse rates. Of 38 patients, 34 (89%) completed the programme, with discontinuations mainly due to anxiety, trauma history, or intolerable neuropathic pain. The decision to start with a fixed dose of 100 mg morphine orally was based on clinical experience and previous evidence. One study demonstrated that a fixed starting dose as low as 90 mg MED was safe, well tolerated, and associated with fewer high‐intensity withdrawal symptoms, while another concluded that substantial initial dose reductions are feasible and effective in a closely monitored inpatient setting (Bienek et al. 2019; Krumova et al. 2013).

### 4.2 Outcomes After OW

The 84% success rate of complete OW aligns with or slightly exceeds comparable studies (Krumova et al. 2013; Huffman et al. 2017). At 3 months, 61% of patients remained opioid‐free, with relapse rates estimated at 11%–40% due to missing data. Longer follow‐up studies report relapse rates of 30%–40% within 12–24 months (Huffman et al. 2017; Krumova et al. 2013). Predictors of relapse include anxiety, functional impairment, and depression, while lower pain intensity after OW predicts abstinence (Huffman et al. 2017; Krumova et al. 2013). Additionally, depressive symptoms have emerged as significant risk factors for dropout and relapse during and after withdrawal (Heiwel et al. 2011). This is particularly noteworthy given that patients in opioid withdrawal programmes often present with elevated depression and pain scores upon admission (Townsend et al. 2008). Interestingly, the initial opioid dose does not seem to correlate with relapse risk, suggesting other factors may play a more critical role (Huffman et al. 2013). These findings highlight the need for effective post‐intervention strategies.

Despite undergoing OW, the average pain intensity in our cohort remained unchanged, with 12 patients (35%) experiencing substantial pain relief and only 3 patients (9%) reporting a significant increase in pain. These seemingly counter‐intuitive results are supported by an electronic database analysis from New Zealand and Australia involving 10,302 patients across 67 pain services (Tardif et al. 2021). In this cohort, 6340 individuals were opioid users with an average daily oral morphine equivalent dose of 56.3 mg (SD, 75.3 mg). Of these, 1724 participants discontinued opioid use and demonstrated post‐treatment improvements in pain experience similar to those of the non‐opioid user group (pain severity decreased from 6.1 [SD, 1.7] to 4.9 [SD, 2] vs. 5.8 [SD, 1.7] to 4.9 [SD, 2]). Furthermore, a recent multicenter randomised controlled trial demonstrated that opioid discontinuation is not necessarily linked to an increase in pain intensity (Sandhu et al. 2023).

One plausible explanation for the observed stable or decreasing pain intensity following OW is the reduction of opioid‐induced hyperalgesia (OIH), a paradoxical condition characterised by increased pain sensitivity resulting from prolonged opioid exposure. The occurrence of OIH is influenced by factors such as the specific opioid used, the dosage, and the duration of exposure (Sampaio‐Cunha and Martins 2022). Preclinical studies have demonstrated that OIH can manifest within 2–3 days (Delgado et al. 2023; Yamakita et al. 2023) or even within hours of opioid administration (Mauermann et al. 2016). This phenomenon likely contributes to the stable pain intensity ratings observed in our study population following withdrawal.

### 4.3 Psychological Impact of Opioid Withdrawal and Adverse Events

The psychological impact of OW is significant, with serious risks like suicidality observed in patients with opioid use disorders (Pfeifer et al. 2021), and there is strong evidence that chronic pain itself is a risk factor for suicidality (Racine 2018). While the post‐discharge suicide in our cohort may not directly link to OW, it underscores the need for robust psychological support, particularly for patients with pre‐existing depression or psychiatric conditions. Given the unpredictability of suicidal ideation, future protocols should incorporate comprehensive follow‐up care tailored to identify and mitigate the risks associated with the recovery process.

Recent research has highlighted that patients who discontinue opioids may experience a recurrence or worsening of mental health issues, especially when the motivation to withdraw is externally driven (e.g., by the healthcare system or clinicians) (Yarborough et al. 2024). This phenomenon may be attributed to the overlap between opioid action and emotion regulation within the central nervous system (Nummenmaa and Tuominen 2018). Opioids modulate not only pain intensity but also pain‐related unpleasantness, which could explain why individuals with a high emotional burden—potentially stemming from early life stress or trauma—might resort to opioid use as a form of self‐medication (Kimmey et al. 2022; Oswald et al. 2021). This suggests that psychological suffering, rather than just pain intensity, may drive opioid use and complicate withdrawal efforts.

Therefore, future studies should consider including trauma‐related and emotion regulation assessments at baseline to better understand the role of psychological factors in opioid dependence and withdrawal outcomes. These assessments could provide deeper insights into the mechanisms driving opioid use and help in developing more targeted interventions, thereby improving the success rates of OW programmes and reducing the risk of adverse outcomes.

To ensure comprehensive monitoring and management of medical issues, OW was conducted at the Department of Internal Medicine. Thanks to the controlled clinical inpatient setting, no serious medical side effects occurred during hospitalisation, and there were no incidents of overdose.

### 4.4 Strengths and Limitations

This study was designed as a quality control initiative, systematically collecting prospective data to assess the feasibility of a structured inpatient OW programme. Despite the small sample size, our heterogeneous patient cohort reflects real‐world clinical practice, encompassing a range of pain conditions and medication histories.

A key factor in the programme's success was patient motivation, which plays a critical role in opioid discontinuation. Patients who recognise the risks of continued opioid use may exhibit a stronger readiness for withdrawal (Crouch et al. 2023), while external environmental cues can trigger cravings, complicating long‐term abstinence (Frenois et al. 2005). Such mechanisms underscore how opioid withdrawal outcomes are shaped by both intrinsic patient characteristics and external stimuli.

The programme's resource‐intensive nature and strict selection criteria, combined with the impact of the COVID‐19 pandemic, contributed to the limited cohort size (42 patients over 5 years). Careful patient selection was necessary to prioritise those most likely to benefit, particularly high‐dose opioid users who may not succeed with outpatient withdrawal. While this restricts generalisability, it enhances internal validity, ensuring the feasibility of withdrawal in a controlled setting.

The cost of the intervention, including Phase 1 and Phase 2, amounted to approximately CHF 35,550 per patient. This upfront investment must be weighed against the substantial cost of prolonged opioid therapy. For example, one patient previously required CHF 14,000 per month in opioid‐related expenses, resulting in annual costs of approximately CHF 150,000. Following successful withdrawal, these expenses were eliminated, demonstrating the potential economic benefit of structured inpatient withdrawal for select high‐cost cases.

Scalability remains a challenge. While many patients on lower opioid doses can successfully taper in outpatient settings, an inpatient approach remains clinically and economically justified for selected high‐dose, complex cases. Future research should explore cost–benefit analyses and alternative withdrawal models, such as hybrid inpatient–outpatient programmes, to improve accessibility while maintaining effectiveness.

The absence of a control group prevents direct attribution of outcomes to the intervention. Additionally, missing baseline and psychometric data limit the ability to fully assess psychological predictors of OW success. While adherence was high, with only four dropouts, one‐fifth of patients had incomplete follow‐up data, restricting further analysis of additional important pain subdomains and psychosocial changes.

Future studies should focus on long‐term outcomes, particularly factors influencing relapse, loss to follow‐up, and emotional challenges post‐withdrawal. Understanding these variables will be essential in refining OW protocols and optimising long‐term success for chronic pain patients.

## 5 Conclusion

This study confirms the feasibility and short‐term efficacy of a novel two‐phase opioid withdrawal (OW) programme, with 84% of participants successfully discontinuing opioids by the end of the programme. While most patients maintained stable pain intensity, a considerable proportion experienced a clinically meaningful reduction in pain. However, the occurrence of serious adverse events, including suicide, highlights the inherent risks associated with OW. These findings underscore the critical need for meticulous patient selection, comprehensive psychological support, and ongoing monitoring to ensure patient safety and optimise long‐term outcomes.

## Author Contributions

Study conception and design: Konrad Streitberger, Maria M. Wertli. Acquisition of data: Konrad Streitberger, Maria M. Wertli, Nina Bischoff, Kyrill Schwegler, Christine Baumgartner, Nora Sutter, Larissa T. Blättler, Anna Saliba, Michael A. Harnik. Analysis and interpretation of data: Michael A. Harnik, Anna Saliba, Larissa T. Blättler. Drafting the manuscript and revising it critically: all authors. Approval of the final version: all authors. All authors agree to be accountable for all aspects of the work and to ensure that questions related to the accuracy or integrity of any part of the work are appropriately investigated and resolved.

## Conflicts of Interest

Prof. Streitberger receives funding from Health Promotion Switzerland for the Prevention of Pain Chronification (PrePaC) project.

## Supporting information


Data S1.

## Data Availability

Upon reasonable request.
