# Supplementary material for: Two‐Phase Inpatient Withdrawal Programme for Long‐Term Opioid Use in Non‐Cancer Pain
Source: Eur J Pain. 2025 Mar 21;29(5):e70010. doi: 10.1002/ejp.70010 (PMC11926771; doi:10.1002/ejp.70010)
Supplement: Supplementary file 1 — Data S1. [file EJP-29-0-s001.pdf]

Supplementary Table S1: Stepwise opioid reduction protocol

Day	Dose reduction	Average morphine equivalent dose (mg/day)
1	0% (Initial dose)	Up to 100 mg
2-3	40 - 60%	60 mg
4-5	75 - 85%	30 mg
6-9	Up to 95%	5 - 10 mg
10+	Complete discontinuation	0 mg

The initial opioid therapy was converted to oral morphine solution (Morphini HCL Insel Lösung 20 mg/ml) for precise dose titration. Patients received their dose divided into 6 daily administrations to maintain stable plasma levels. The maximum initial dose was typically set at 100 mg/day, with adjustments based on individual patient needs.

Supplementary Table S2: Additional questionnaires

Variable	Baseline (n = observed patients)	After 3 months (n = observed patients)
PCS (median [IQR])	23 [16; 30], (n = 11)	20 [9; 24], (n = 21)
MPI (median [IQR])	63 [51; 76], (n = 11)	66 [36; 76], (n = 21)
BDI (median [IQR])	7 [4; 11], (n = 13)	5 [2; 9], (n = 21)
**PGIC (Pain), n (%)**		**n = 20**
Very much improved		2 (10%)
Much improved		3 (15%)
Minimally improved		5 (25%)
No change		3 (15%)
Minimally worse		1 (5%)
Much worse		4 (20%)
Very much worse		2 (10%)
**PGIC (Overall Situation), n (%)**		**n=21**
Very much improved		3 (14%)
Much improved		9 (43%)
Minimally improved		2 (10%)
No change		4 (19%)
Minimally worse		0 (0%)
Much worse		2 (10%)
Very much worse		1 (5%)

Data of all included patients (n = 38) are shown as median [Q1; Q3] for continuous or ordinal data and as absolute and relative frequencies (n, %). Observed patient numbers (n) are indicated for each variable and timepoint. PGIC scores (Pain and Overall Situation) were assessed only at the 3-month follow-up. Abbreviations: PCS, Pain Catastrophizing Scale; MPI, Multidimensional Pain Inventory; BDI, Beck Depression Inventory; PGIC, Patient Global Impression of Change.
